# Brain mechanisms underlying automatic and unconscious control of motor action

**DOI:** 10.3389/fnhum.2012.00265

**Published:** 2012-09-26

**Authors:** Kevin D'Ostilio, Gaëtan Garraux

**Affiliations:** ^1^MoVeRe Group, Cyclotron Research Center, University of LiegeLiege, Belgium; ^2^Department of Neurology, University Hospital CenterLiège, Belgium

**Keywords:** subliminal, priming, unconscious, inhibition, volition, motor control, Parkinson disease, neuroimaging

## Abstract

Are we in command of our motor acts?The popular belief holds that our conscious decisions are the direct causes of our actions. However, overwhelming evidence from neurosciences demonstrates that our actions are instead largely driven by brain processes that unfold outside of our consciousness. To study these brain processes, scientists have used a range of different functional brain imaging techniques and experimental protocols, such as subliminal priming. Here, we review recent advances in the field and propose a theoretical model of motor control that may contribute to a better understanding of the pathophysiology of movement disorders such as Parkinson's disease.

## Introduction

In daily life, we usually have the feeling that we are the authors of the actions we make, that the decisions we make and the corresponding movements we perform are consciously initiated and controlled. The belief that our actions are caused by our mental states, and these mental states are causally independent from brain processes reflects a dualistic philosophy (Descartes, 1641). However, the current scientific view holds that human actions and mental states are both biologically determined and stem from patterns of neural activity in the brain. Philosophers and scientists have wondered for centuries about the extent of determinism in our behavior. For example, Freud (1900) highlighted the importance of the unconscious on our wishes and acts. Even if the Freudian unconscious seems today inconceivable, the concept of cognitive unconscious is in the heart of the debates. That is the reason why certain authors call into question the causal role of consciousness in voluntary action (Libet et al., 1983) or even the separation between automatic and controlled behavior, suggesting that automatic and unconscious processes can form an intrinsic part of all behaviors, even the most complex (Sumner and Husain, 2008). Therefore, this review aims to summarize the advances in understanding implicit mechanisms of motor decision and their underlying neural substrates by examining recent research on motor awareness and subliminal priming. In addition to their philosophical ramifications, these findings also have important clinical implications, and may help us better understand and treat motor control disorders such as the Parkinson's disease (PD).

## Voluntary action is unconsciously generated

**Figure 1:**
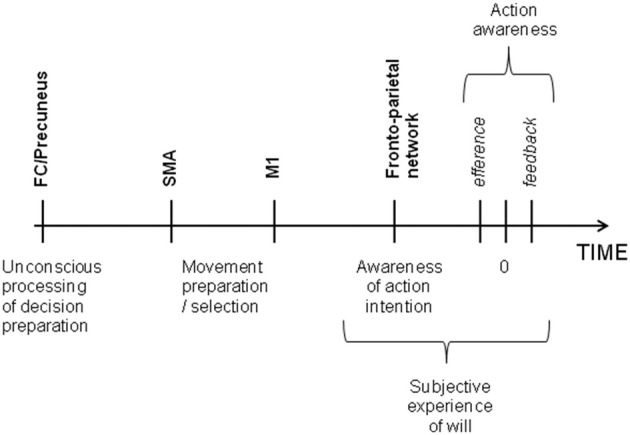
**Time of voluntary initiation of action and the associated brain regions.** Frontal and parietal cortex work together for deciding the action to plan and are also involved in the awareness of motor intention whereas the SMA translates the first unconscious decision into movement. The subjective experience of will comes from the awareness of intention itself, the efferent signal from motor cortex to muscles and feedback sensorial signals.

The idea that intention is a direct translation of desires and goals into behavior is deeply embedded in our culture. But it is supported by experimental evidence? That is the question which interested Benjamin Libet 30 years ago (Libet et al., 1983). In his pioneer experiment, participants were asked to make a voluntary movement at will and to report the exact time on a clock at the instant they had decided to move, while their readiness potential, a change in electroencephalography (EEG) activity over the motor cortex that occurs prior to voluntary movement, was being recorded. The results showed that the preparatory motor activity began more than 350 ms before subjects became aware of the decision to act. More recently, Soon et al. (2008) used a brain decoding statistical method to show that an action could be predicted by blood-oxygen-level-dependent functional magnetic resonance imaging (BOLD fMRI) signal. Although the volunteers felt they consciously decided to move, the vector machine could classify the outcome of their decision by means of the activity in several cortical regions, such as the precuneus and the fronto-polar cortex, up to a few seconds before the decision to move entered awareness, while the activity in the supplementary motor area (SMA) determined the timing of that decision. Others experiments have shown that a large fronto-parietal network was involved in the conscious experience of decision to make a voluntary movement (Lau et al., 2004; Sirigu et al., 2004). This conscious experience of motor intention could be influenced by efferent signals (i.e., premotor activity) and sensory feedback signals (i.e., feedback from the movement itself) (Obhi, 2007; Strother and Obhi, 2009). Brain stimulation techniques have also brought us new insights into the brain processes underlying the subjective experience of will. In 1969, Delgado directly stimulated the internal capsule, inducing head turning and body movements in the patient, which the patient experienced as spontaneous and which he fought to control (Delgado, 1969). The arrival of transcranial magnetic stimulation (TMS), which is non-invasive, allowed scientists to modulate the brain processes underlying the experience of voluntary control in large samples of healthy subjects. For example, Brasil-Neto et al. (1992) demonstrated that a choice between two alternatives could be bias by the stimulation of the motor cortex without disrupting the conscious perception of volition. Taken together, these experiments provide clear evidence suggesting that a voluntary action might be initiated unconsciously and the movement selection always precedes awareness while the intention feeling comes afterward (Wegner and Wheatley, 1999) According to the model considering free will as a perception (Hallett, 2007), intention does not cause our actions, but emerges as a consequence of brain processes underlying movement preparation. More specifically, a study by Desmurget et al. (2009) suggested that motor intention was a consequence of increased parietal cortex activity during motor preparation prior to motor execution. In their study, Desmurget et al. showed that stimulating the posterior parietal cortex of patients could induce a strong reportable intention and desire to move. This finding corroborates the neuroscientific view of biological determinism and is also not contradictory with the idea that conscious will is an illusion. Even if stimulating parietal cortex can create a motor intention at a time *t*, it cannot be taken as a proof that it was not preceded by an unconscious decision phase at a time *t*-1 (see Figure 1).

Additional evidence of the importance of the unconscious cognitive processes in voluntary motor action comes from research employing subliminal priming, which suggests that a conscious decision can be affected by stimuli that were never consciously perceived. We review this evidence in the next section.

## Voluntary action is modulated by subliminal stimuli

In the 1970 s, researchers discovered that a patient with a primary visual cortex lesion could continue to accurately differentiate between two visual stimuli in a motor task despite not being able to consciously perceive these stimuli (Weiskrantz et al., 1974) This surprising phenomenon was termed “blindsight” and interpreted as evidence of a direct perceptuo-motor link which allowed visual information to directly activate associated motor responses, without any conscious awareness of this visual information (Neumann, 1990). Experiments in healthy subjects confirmed this hypothesis. Indeed, Neumann and Klotz (1994) showed that reaction time and accuracy of a target response could be modulated by a subliminal prime, such that a prime compatible with the response enhanced this response, whereas a prime incompatible with the response impaired the response. This compatibility effect had also been reported when the relation between the prime and the target was semantic (Dehaene et al., 1998).

The experiments reviewed above demonstrated that motor actions can be influenced by external stimuli that are not consciously perceived. However, these experiments do not allow determining which processing level is reached by unconsciously perceived stimuli. This issue was addressed by recording brain activity changes in response to external stimuli that were not consciously perceived. Dehaene et al. (1998) were among the first to show that the effect of a subliminal visual stimulus on brain activity could spread up to the level of brain areas involved in motor response programming and execution. However, this result in motor-related areas was not easy to interpret because the subliminal stimuli were systematically followed by a motor response. Many studies provided evidence supporting a direct perceptuo-motor link for consciously perceived stimuli: simply viewing an object can prime manual responses that might be used to grasp the object, even when the person has no intention of making the associated movement (Tucker and Ellis, 1998; Grezes and Decety, 2002; Derbyshire et al., 2006). Recently, our group (D'Ostilio and Garraux, 2011), have reported new fMRI evidence that such automatic and unconscious activity in motor-related brain areas can be induced in a subliminal masked prime task with no-response stimuli, when no movement is executed. In this fMRI study, healthy volunteers performed a subliminal masked prime task, in which they had to respond by pressing a left or right button depending on the direction of the arrow targets. In 40% of trials, a no-response target was displayed and subjects were instructed to simply watch the middle of the screen without making any response. A subliminal stimulus that could be either an arrow (compatible or incompatible with the target) or a neutral stimulus (× sign) was presented on all trials. fMRI analyses of these no-response trials revealed activation in the medial premotor areas, including the SMA, when subliminal arrow stimuli were presented compared to neutral subliminal stimuli.

The effect of external stimuli that are not consciously perceived on voluntary motor action may also involve higher level areas. By using subliminal priming paradigm, several new studies demonstrated that certain executive functions, such as task switching, inhibition or error monitoring for example, could be unconsciously modulated by certain brain regions traditionally associated with conscious cognitive control (Lau and Passingham, 2007; Pavone et al., 2009; for a review see also Van Gaal et al., 2008, 2010, 2012). In a recent experiment (D'Ostilio and Garraux, 2012), we corroborated this hypothesis by showing that subliminal motor priming was underlain by activity changes in frontal areas. Whereas unconscious facilitation was related to reduce activity in the motor-related areas, conflict induced by a subliminal prime activated regions involved in high-level motor control, traditionally linked to the cognitive system, such as the anterior cingulate cortex and the dorsolateral prefrontal cortex. As a corollary, these results call into question the traditional theories considering consciousness and cognitive control as being intimately related, as well as the exclusive involvement of prefrontal cortex in conscious processing of information.

As our behavior is flexible, the automatic activation of corresponding motor plans should be suppressed if the movement is not intended to be immediately executed. In 1998, Eimer and Schlaghecken provided evidence supporting the existence of this inhibitory process by administering a specific subliminal masked prime task to a group of healthy subjects. For this task, participants were asked to make speeded button presses with the left or right hand following leftward or rightward pointing arrows, which were preceded by a subliminal masked prime arrow. By manipulating the interstimulus interval (ISI), i.e., the interval between the mask and the target (80 ms <ISI <200), they observed a Negative Compatibility Effect (NCE), namely a performance cost for compatible trials (longer reaction times, more errors) and a performance benefit for incompatible trials (shorter reaction times, fewer errors). This effect was interpreted as an index of the automatic motor self-inhibition mechanism that suppresses the partial motor activation caused by the prime. Indeed, this self-inhibition produces a response conflict in the compatible condition, subjects having more difficulties to answer to an arrow which was beforehand suppressed. In the same study, this reversal effect was validated by the EEG activity of motor cortex, the Lateralized Readiness Potential (LRP), an electrophysiological index of unimanual response preparation. The LRP revealed an initial activation of the response corresponding to the prime followed by a polarity shift, namely an activation of the opposite response, reflecting the inhibition of the initial response tendency (Eimer and Schlaghecken, 1998, 2003; Praamstra and Seiss, 2005). According to Libet's experiment, subliminal primes also have the power to influence free choices between response alternatives (Schlaghecken and Eimer, 2004; Kiesel et al., 2006), suggesting again that the initiation of a voluntary action relies on unconscious processes.

The model proposed by Eimer and Schlaghecken (2003) has generated a series of experiments aimed at identifying the neural correlates of the automatic and unconscious inhibition especially by using a lesional approach. Sumner et al. (2007) suggested a key role of the medial frontal cortex in the automatic suppression of unwanted motor plans. They administrated the subliminal masked prime task to two patients with small lesions of the SMA or the supplementary eye field (SEF). These patients produced opposite-to-normal effects, namely a facilitation effect instead of inhibition and this occurred in an effector-specific manner mirroring the extent of the patients' damage. Sub-cortical regions were also associated with these automatic and unconscious processes, notably the basal ganglia and thalamus (Aron et al., 2003). Aron et al. (2003) administered the subliminal masked prime task to 15 patients with genetically-confirmed Huntington's disease, a progressive neurodegenerative disease characterized by nerve cell loss in the striatum, leading to chorea, a hyperkinetic movement disorder. Both controls and patients showed a NCE of equal magnitude. According to chorea severity, the researchers observed a bimodal distribution, namely an absence of NCE in patients with greater chorea and a stronger NCE than controls in patients with less chorea. In the same study, the authors also used fMRI as healthy volunteers performed the same task and found activity changes in the caudate and thalamus for the inhibition condition, an observation that is in agreement with their findings in Huntington patients. This pattern of brain regions involved can lead us to rethink some motor control diseases as an impairment of theses automatic and unconscious primary processes.

## A new look on motor control disorders

### Frontal patients

Several disorders of motor control are related to disturbances in fronto-striatal circuits that may result from an impairment of automatic and unconscious processes such as motor activation and inhibition. For instance, the alien hand syndrome, often characterized by unwanted movements that arise without any sense of volition, with the patient feeling that his or her hand does not belong to them. The affected hand can grasp a nearby object and have difficulties to release it or even violently throw it. This syndrome is often linked with a damage to the medial frontal lobe or the corpus callosum (Gasquoine, 1993; Kertesz and McMonagle, 2010). The fact that some patients think they do not have the intention to cause the action they make and inversely that a brain stimulation of a movement can produce a feeling of will or that the brain of choreic Huntington's disease patient sometimes interprets the involuntary movements as being voluntary (Hallett, 2007), provide examples supporting the view of an unconscious causation of action. Furthermore, this dissociation between the conscious decision to act and the action itself is in line with the idea that an inhibitory control could be triggered unconsciously. Thus, in those patients, an internal or environmental stimulation could produce a representation and an activation of the associated movement but could not be automatically suppressed, even when the action must not be performed. The impairment of these primary mechanisms could also explain the disinhibition problem of utilization behavior, a related syndrome in which the patient could reach out and automatically use objects in his environment, the act being often inappropriate to the context, like picking up a toothbrush and brushing his teeth in response to a toothbrush being placed in front of him, even if he is in a friend's house (Archibald et al., 2001).

### Basal ganglia disorders

Basal ganglia circuits are sites of interaction between motor, cognitive, and limbic systems that play an important role in the control of movement planning and execution, notably in initiating, inhibiting, and switching behaviors, as well as in the processing of reward and other feedback (Aron et al., 2009). The study of PD, a progressive neurodegenerative basal ganglia disorder characterized by hypokinetic motor symptoms such as akinesia, offers a unique opportunity to study the automatic and unconscious processes which modulate action control. For that purpose, Seiss and Praamstra (2004) administrated the masked prime task to PD patients on their usual dopaminergic medications and showed a delay in the NCE although the overall response time course was normal (Seiss and Praamstra, 2006), suggesting that a part of automatic motor processes are probably preserved in PD. Furthermore, the motor control impairment in PD is interesting because of their kinesia paradoxica. For example, akinetic patients in instances of emergency may be able to make quick movements such as suddenly running. This can suggest that patients with PD have intact motor programmes but are usually unable to recruit them adequately (Jankovic, 2008). Mazzoni et al. (2007) proposed that the slowness of movement in PD may be attributable to the poor motor motivation caused by a shift in the cost/benefit ratio of moving fast, as a result of loss of dopamine, this neurotransmitter being also linked to reward mechanisms (de la Fuente-Fernandez et al., 2002). Thus, we can hypothesize that the automatic and unconscious motor activation/inhibition processes are dependent on the dopamine circuit, and so of the motivation to realize a given action. According to the proposed model, the system normally chooses to suppress an activated movement, if and only if this movement is not reinforced within 100–200 ms, whereas if a valuable stimulus is presented to the system, the initiated movement is executed. The existence of an unconscious motivation system was underlined by Pessiglione et al. (2007) who demonstrated, by using an incentive force task with money as a subliminal reward, that even when participants could not report the amount of money at stake, they deployed a greater force for high reward. In addition, they localized this subliminal reward expectation system in basal ganglia, more precisely in the ventral pallidum. Furthermore, Schmidt et al. (2008) reported that patients with bilateral striato-pallidal lesion failed to integrate the affective value of reward into their motor behavior. Taken together, this research can help us to reinterpret basal ganglia motor disorders like PD, as an unbalance between automatic activation and inhibition processes, monitored by a basal ganglia motor motivation system.

## Conclusions

Although many theoretical models have been developed to explain aspects of motor control, the mechanistic basis of motor control remains incompletely understood. In this review, we attempted to review recent research on the role of automatic and unconscious processes in the voluntary control of motor action. We first focused on the role of unconsciousness in action preparation, suggesting that the feeling of motor intention comes afterward without directly causing behavior. Next, we reviewed growing evidence that unconscious and automatic processes play an important role in both activation and inhibition of motor behaviors. Such processes could be present in all behavior and could be specially revealed by brain-lesioned patients who manifest difficulties controlling the actions they intend or do not intend to execute. In addition, we introduce a new hypothesis which may explain the diversity of motor control diseases, namely an unbalance between the primary self-activation, self-inhibition mechanisms, and the unconscious motivation to act.

### Conflict of interest statement

The authors declare that the research was conducted in the absence of any commercial or financial relationships that could be construed as a potential conflict of interest.
